# Mortality, fecundity and development among bed bugs (Cimex lectularius) exposed to prolonged, intermediate cold stress

**DOI:** 10.1002/ps.4504

**Published:** 2017-01-31

**Authors:** Bjørn A Rukke, Morten Hage, Anders Aak

**Affiliations:** ^1^ Department of Pest ControlNorwegian Institute of Public Health, NydalenOsloNorway

**Keywords:** bed bug, cold treatment, mortality, fecundity, development, IPM

## Abstract

**BACKGROUND:**

Bed bugs (Cimex lectularius L.) have returned as a nuisance pest worldwide. Their ability to withstand different types of environmental stress should be explored in order potentially to increase the efficiency of control methods.

**RESULTS:**

Immediate and long‐term effects of exposure to temperatures from 0 to −10 °C for 1, 2 and 3 weeks are reported. Fifth‐instar nymphs and adults were exposed to constant or fluctuating temperatures. Increased cold and extended time yielded higher mortality; nymphs were more resilient than adults at the shorter durations of exposure. At intermediate temperatures, mortality was higher at constant compared with fluctuating temperatures, whereas all individuals died after 3 weeks of exposure to −7 °C. The success among survivors after cold treatment was also affected in terms of reduced egg production, hatching success and the ability of fifth‐instar nymphs to advance into the adult stage; however, nymphs produced after cold treatment developed normally.

**CONCLUSIONS:**

Detrimental effects of prolonged exposure to low temperatures were seen in bed bugs both during and after cold treatment. The results suggest that temperatures below −7 °C can be applied by laymen to control this pest in small items if available treatment time is of less concern. © 2016 The Authors. *Pest Management Science* published by John Wiley & Sons Ltd on behalf of Society of Chemical Industry.

## 1 INTRODUCTION

Bed bugs (*Cimex lectularius* L.) have been a nuisance to man worldwide since early history. They have been controlled for many years through the use of pesticides, but recently these nuisance pests have become prolific again. Resistance to pesticides and increased travel are main contributors to the resurgence. These pests cause both clinical problems and control challenges.^1^, ^2^, ^3^ Eradication is often difficult and costly, and integrated pest management (IPM) approaches are viewed as obligatory for efficient handling of infestations.^2^, ^4^ Currently, the bed bug situation is out of control in many places, and preventing their introduction into buildings and apartments in new areas is crucial in the attempt to limit their worldwide increase.

Deep‐freezing below −30 °C for a few days is sufficient to treat infested items, such as luggage, clothes and furniture, and is used frequently by pest controllers. These low temperatures are not available to laymen who only have access to home freezers or, in some geographical areas, low outdoor winter temperatures. Home freezers rarely drop below −20 °C and may fluctuate into less severe temperature intervals.^5^ To ensure 100% mortality using these facilities, the length of exposure has to be extended. Fortunately, in this respect the survival of insects during moderate levels of thermal stress is mostly a response to the duration of the stress.^6^, ^7^ It is thus possible that temperatures considered harmless to bed bugs during brief exposure may become fatal if treatment time is extended.

Neither cold acclimation at 4 °C for 2 weeks nor dehydration (15% loss of water content) has been shown to enhance cold tolerance in bed bugs.^8^ A 100% mortality rate in adults has been reported after 1 h at either −16 °C or −18 °C, depending on the absence or presence of rapid cold hardening,^8^ or at −17 °C after 2 h without cold hardening.^9^ Olson *et al*.^5^ point to some stronger resilience through experiments and subsequent modelling that indicate a tolerance of up to 72 h at −18 °C. They also estimate that survival in some individuals is more than 1 week at −12 °C. Specific information on bed bug survival after more than 1 week of exposure to temperatures between 0 and −10 °C is missing, and the impact of temperature fluctuations during exposure is also incompletely described.

Cold is known to influence insect survival but may also affect other essential biological functions. Insect species investigated for prolonged exposure to sublethal cold have developmental abnormalities,^10^, ^11^ loss of coordination^12^ and reduced fecundity.^13^ More factors than direct mortality should thus be included as experimental parameters to provide a more complete picture of the consequences of cold treatment for population dynamics.^7^ Detrimental maternal effects are known among bed bugs surviving long periods of sublethal heat stress,^14^ and similar consequences may be suspected at the other end of the temperature scale.

The present study explores the effects of 1–3 weeks of exposure of bed bug nymphs and adults to constant and fluctuating temperatures ranging from 0 to −10 °C. We report overall mortality as well as additional effects such as fecundity and development on the survivors and their progeny.

## 2 EXPERIMENTAL METHODS

### 2.1 Experimental insects

Bed bugs originated from two hotels located in Oslo, Norway, and founder individuals were collected in 2009. They were fed human blood artificially through parafilm blood bags.^15^ Cultures were maintained in climate chambers (Sanyo – MLR‐351H; Medinor ASA, Oslo, Norway) with a 16/8 h light/dark cycle at 22 °C and 60% relative humidity. Fourth‐ and fifth‐instar nymphs were collected, fed and allowed to moult during the following fortnight to provide newly emerged fifth instars and adults to be used in experiments.

### 2.2 Experimental protocol

Newly emerged adults were collected and fed. Only fully engorged adults were selected and sorted into 140 mL polyethylene boxes with a single 2 × 2 cm paper towel sheet. In each box, we placed three males, three females and six newly emerged, unfed fifth‐instar nymphs. For each cold treatment, we used eight boxes with a total of 96 bed bugs. The boxes were then kept at 22 °C and 60% relative humidity for 7 days. On day 8, the boxes were placed at 0 °C for 1 h to induce rapid cold hardening,^8^ and then moved into cooling incubators (Sanyo – MIR 153 or Binder – KB115; Medinor ASA, Oslo, Norway), maintaining their respective cold treatment temperatures. After the cold treatment period, the boxes were transferred back to the climate chamber with 22 °C and 60% relative humidity. Mortality was checked after 24 h at 22 °C and rechecked after 7 days to avoid overestimation of mortality due to temporary knockdown. The survivors were reorganised and relocated to new boxes with three males, three females and six nymphs to check for long‐term survival, development of fifth‐instar nymphs, egg production and nymphal development. During the following 8 weeks, the bed bugs and their progeny were fed each fortnight (supporting information Fig. S1). Hence, fifth‐instar nymphs and adults were allowed to feed 4 times, while produced nymphs were able to feed 0–3 times, depending on when they hatched during this period. Fourteen days after the last feeding, the experiment was terminated, and the number of eggs and individuals of different stages was registered.

### 2.3 Cold treatments

Bed bugs were either exposed to constant temperatures of 0, −5, −7 or −10 °C for 1, 2 or 3 weeks or to fluctuating temperatures providing the same average temperatures within the equivalent time periods (supporting information Fig. S2). In the fluctuating treatments, they experienced a 24 h cycle with 8 h at 4 deg above and 16 h at 2 deg below the assigned treatment temperature (example of −5 °C given in supporting information Fig. S1). Bugs in eight boxes maintained constantly at 22 °C and subsequently fed in parallel with cold‐treated insects were used as a control for mortality and for comparison of life history traits among survivors. We also had a negative control that experienced 1 week cold treatment at −15 °C to confirm the predicted 100% mortality.

### 2.4 Statistical analyses

The data were analysed using SigmaPlot v.12.3 (Systat Software, San Jose, CA). Multiple comparisons were performed using analysis of variance (ANOVA) followed by Tukey tests or the Holm–Sidak method, and pairwise comparisons were performed using paired *t*‐tests. The level of significance was set to 0.05. If test for normality failed, we used the non‐parametric alternatives, the Kruskal–Wallis ANOVA or the Wilcoxon signed rank test. High mortality at some of the more severe treatments reduced the number of available bed bugs, allowing only a small number of complete reorganised boxes. The minimum number of boxes needed for inclusion of a treatment in statistical analyses was set at four.

## 3 RESULTS

### 3.1 Survival of bed bugs during cold treatment

All control bed bugs at 22 °C survived, whereas for those kept at −15 °C for 1 week, mortality was 100%. In between these two extremes, the fifth‐instar nymphs had higher survival than the adults (Wilcoxon signed‐rank test: *Z* = 3.040, *P* <0.001), and consequently the two stages were analysed separately.

**Figure 1:**
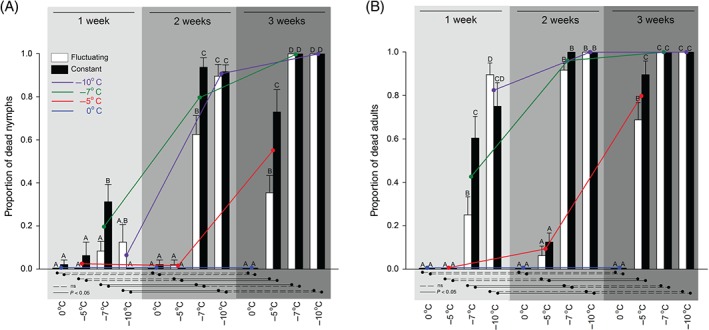
Average ± SE mortality of fifth‐instar nymphs (A) and adult C. lectularius (B) after cold treatment. Different letters above bars show a significant difference (Tukey's test, P < 0.05) between the eight temperature treatments within the same treatment length (1, 2 or 3 weeks). Lines below bars show a significant difference (Tukey's test, P < 0.05) in mortality between the different treatment lengths (1, 2 or 3 weeks) of the same temperature treatment. Coloured lines show the averaged value of fluctuating and constant treatment of each temperature over different treatment lengths (1, 2 and 3 weeks).

We observed an overall effect on survival from both time and temperature, and the interaction between the two factors in both nymphs and adults was significant (Fig. 1 and Table 1). Trends, interactions and elevated cold resilience in nymphs are indicated by the coloured lines in Fig. 1, where corresponding constant and fluctuating treatments have been averaged to ease interpretation. Most bed bugs survived at 0 °C, irrespective of exposure time. At −5 °C, a strong increase occurred in mortality when extending the duration of the exposure from 2 to 3 weeks, with a more substantial increase in mortality occurring among adults (9 to 79%) than among nymphs (1 to 54%). At −7 °C and −10 °C, there was a distinct increase in mortality when cold exposure was extended from 1 to 2 weeks, and 100% mortality was achieved after 3 weeks at both temperatures. After 1 week treatment at these two temperatures, the nymphs had less than 20% mortality compared with more than twice as much among adults (43 and 82% respectively).

**Table 1:** Survival of C. lectularius fifth‐instar nymphs and adults after cold treatment

Source	df	Nymphs^a^				Adults^b^			
		Sum of squares	Mean square	*F*	*P*	Sum of squares	Mean square	*F*	*P*
Length	2	10.25	5.12	301.31	<0.001	4.76	2.38	153.21	<0.001
Temp.	7	16.39	2.34	137.66	<0.001	27.90	4.00	256.78	<0.001
Length × temperature	14	7.29	0.52	30.61	<0.001	5.25	0.38	24.17	<0.001
Error	168	2.86	0.02			2.61	0.02		
Total	191	36.79	0.19			40.52	0.21		

^a,b^ Two‐way ANOVAs describe the effect of the two predictor variables, treatment length and treatment temperature.

Differences in temperature regimen (constant or fluctuating) had a significant impact during intermediate thermal stress only. Nymphs and adults exposed to −7 °C for 1 week or to −5 °C for 3 weeks and nymphs exposed to −7 °C for 2 weeks had higher mortality at constant compared with fluctuating temperatures (Fig. 1).

### 3.2 Survival, development and maternal effects after cold treatment

There was no difference in fifth‐instar moulting, egg production, hatching success or moults per nymph (paired *t*‐test: fifth‐instar moulting, *P* =0.973; egg production, *P* = 0.773; hatching success, *P* = 0.147; moults per nymph, *P* = 0.610) between bed bugs exposed to fluctuating or constant temperatures of the same time–temperature combination, nor did any adult mortality occur for the rest of the experimental period. Therefore, data from corresponding fluctuating and constant treatments were pooled in further analyses.

**Figure 2:**
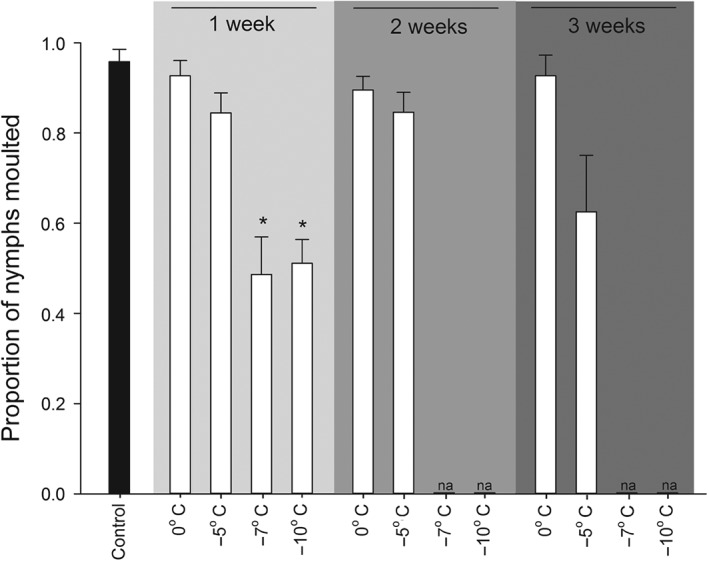
Average ± SE proportion of cold‐treated C. lectularius fifth‐instar nymphs that moulted to adults during an 8 week period after cold treatment. Only treatments with enough surviving nymphs to make four or more complete boxes with six nymphs each are included in the analyses (N = 4–16 boxes). Other treatments are marked as not applicable (na). An asterisk (*) denotes significant difference in treatments compared with the control treatment of 22 °C (Dunn's method, P < 0.05).

**Figure 3:**
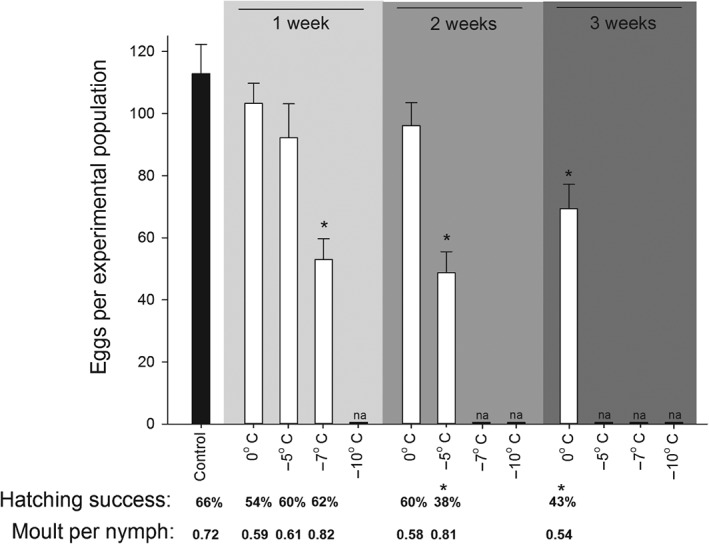
Average ± SE egg production during an 8 week period after cold treatment in C. lectularius experimental populations consisting of three males, three females and six fifth‐instar nymphs. Initial individuals were allowed to feed each fortnight for a total of four feedings, while their offspring were able to feed 0–3 times depending on when they hatched. Also shown is hatching success (number of nymphs divided by number of eggs) in percentage and moult per nymph (number of moults divided by number of nymphs) as a measure of progeny development. Only treatments with enough surviving adults after cold treatment to make four or more boxes are shown (N = 4–16 boxes). Other treatments are marked as not applicable (na). An asterisk (*) denotes a significant difference in treatments compared with the control treatment of 22 °C (Holm–Sidak method, P < 0.05, or Dunn's method, P < 0.05).

The exposure time and temperature of the previous cold exposure influenced moulting ability of surviving fifth‐instar nymphs (Kruskal–Wallis ANOVA on ranks: *H* = 53.789, df = 8, *P* < 0.001), with 1 week of −7 °C and −10 °C exposure having a decreased number of emerged adults compared with the control (Fig. 2). Egg production was also reduced by former cold exposure (ANOVA: *F*_6,85_ = 7.834, *P* < 0.001), as was their hatching success (Kruskal–Wallis ANOVA on ranks: *H* = 29.582, df = 6, *P* < 0.001). These two effects were seen in bed bugs having experienced 3 weeks at 0 °C or 2 weeks at −5 °C only (Fig. 3). Egg production was reduced at exposure to −7 °C for 1 week. Longer and colder treatments killed too many bed bugs to allow for tests of subsequent effects, whereas less severe treatments had no negative effects.

Despite an overall effect in number of moults completed per offspring (Kruskal–Wallis ANOVA on ranks: *H* = 20.233, df = 6, *P* = 0.003), no treatment was found to be different from the control, based on the more conservative *post hoc* tests (Fig. 3). This lack of maternal effect on progeny development is supported by the observation that in all of these treatments some nymphs reached the third developmental stage.

## 4 DISCUSSION AND CONCLUSION

This study shows the complexity of temperature treatment and highlights the need for a broader understanding of the responses to stress inflicted by low temperatures on bed bugs. Increased severity of cold treatment reduced not only bed bug survival but also the future success of survivors through moulting ability and progeny production.

Our adult mortality measurements at 1 week of exposure resembled the sixth day mortality modelled by Olson *et al*.,^5^ which predicts around 20, 60 and 90% death at −5 °C, −7°C and −10 °C. However, our nymph mortality was substantially lower than the predictions made by their merged nymph–adult model. This points to the uncertainty attached to modelling, which may simplify relevant relations and interactions. In the present study we also found that constant and fluctuating regimens produced minor, but noteworthy, differences in mortality. This indicates additional prediction uncertainty in unstable systems. Repeated periods of elevated temperatures, despite being combined with alternating colder periods, seem to relieve some of the thermal stress, as indicated by the lower mortality at fluctuating treatments in some of the less severe time–temperature combinations. This is interesting because some insects have shown improved survival through physiological repair in response to brief fluctuations into higher temperatures (20 °C),^16^, ^17^, ^18^, ^19^ and our study might indicate similar tendencies, even though fluctuations are much smaller and close to or below 0 °C. Yet, the general trends in responses to both fluctuating and constant thermal stress largely concur, and combined with the formerly reported bed bug cold tolerance,^5^, ^8^, ^9^ it becomes evident that the time demanded for efficient cold treatment drops rapidly from −10 °C to −18 °C, and increases to 3 weeks in the range between −7 °C and −10 °C. To obtain 100% bed bug mortality at temperatures above −7 °C, more than 3 weeks are needed and seem consequently of less applied value.

An interesting finding in our study is the difference in cold susceptibility between nymphs and adults. Survival during cold stress has previously been reported to be independent of both stage and feeding status,^5^ and dehydrated (15%) adults did not survive better after 1 h of exposure to temperatures between −10 °C and −16 °C.^8^ Our study design could not separate feeding status from stage effects because adults had been fed 1 week prior to cold exposure, while nymphs were newly moulted, starved for more than 1 week and likely to be more dehydrated. Although these differences are subtle, the probability of nucleation (freezing) of a liquid at subzero temperatures is a function of temperature, solute concentration, volume and time,^7^ and it can be speculated that extended periods of cold stress may result in higher survival among starved nymphs owing to a reduced risk of freezing combined with the expected chilling injuries. Mechanisms behind the detrimental effects of cold treatment were not investigated in this study, but direct chilling injuries due to changes in neuromuscular transmission, decreased enzyme activity, changed protein structure or oxidative stress,^7^, ^20^, ^21^, ^22^, ^23^, ^24^, ^25^ less investigated indirect chilling injuries^7^ and freezing injuries^7^, ^10^, ^25^, ^26^ may have occurred.

It is important to look beyond just current mortality effects and include additional costs in the overall assessments of the cold treatment. The observed effects of moult failure and reduced fecundity may have a large impact on individual fitness, demographic structure and long‐term population development. However, we found no maternal effects, as the F1 generation seemed not to be affected once being able to hatch from eggs. This is different from sublethal heat‐stressed bed bugs, which produced apparently normal hatchlings that were unable to develop beyond the first instar.^14^ The negative effects induced by heat resemble what is seen when the maternally transmitted bacterial *Wolbachia* symbiont is removed,^27^, ^28^, ^29^ while it seems that the symbiont is not severely affected by the present cold treatment regime.

How thermal tolerance tests are performed is essential for the experimental outcome. Cooling and subsequent warming rates may strongly influence cold tolerance in insects^23^, ^30^, ^31^, ^32^, ^33^ and should be considered in an ecological approach to ensure that the full response variation is covered.^7^ However, dealing with pest control and freezing as a tool to prevent infestation is far from a natural setting. In our approach towards practical guidelines, we therefore tried actually to mimic conditions met during management without any long‐term acclimation. We did, however, apply rapid cold hardening^8^ to ensure that our mortality measures were not overestimated, which might eventually contribute to treatment failure due to erroneous tolerance limits.

Efficacy of cold treatments in bed bug management is clearly a function of both temperature and time. We chose the particular time–temperature regimen, because it supplements missing empirical data and probably includes temperatures found in some household freezers and during winter in cold climate areas. Treatment at such temperature regimens will probably not be utilised in professional bed bug management where reduction in treatment time is essential and investment in equipment capable of maintaining temperatures well below −20 °C often will be cost beneficial. However, a do‐it‐yourself solution for treatment of small items such as luggage is clearly possible if temperatures are kept well below −5 °C. Most home freezers should hold an average of well below −7 °C and will consequently demand less than a fortnight for successful treatment. If the freezer fluctuates below the typical target value of −18 °C in some periods,^5^ it will dramatically reduce the time needed for eradication. Outdoor treatment of luggage and furniture may also be within reach in more harsh outdoor winter conditions. Some housing facilities such as simple cabins, holiday cottages, caravans or fisherman's shacks may also utilise low outdoor temperatures during winter in truly cold areas. Regardless of objects treated, temperatures ought to be monitored to detect fluctuations and to pinpoint termination of treatment, because we found temperature fluctuation to reduce overall temperature effects.

Another applied aspect arising from this study is the potential control value achieved from reduced fecundity and developmental abnormality. If cold stress prevents further development in exposed nymphs, potential survivors will not contribute to population recovery. These secondary effects may also contribute in integrated pest management where other control measures, such as pesticides, desiccant dusts and steam, are also used. Additionally, and in terms of the introduction to new locations, seriously cold‐affected colonisers may actually not be capable of initiating new populations, which in itself is the essence of preventive measures.

Cold treatment at temperatures between −5 °C and −10 °C seems to be a minor element in the control options against bed bugs. However, if intense cold is unavailable and treatment time is of a lesser concern, intermediate subzero temperatures can be utilised. Such treatments are applicable in do‐it‐yourself solutions and provide a low‐cost alternative that can prevent bed bug introduction into private homes. Public awareness of the sufficient time–temperature demands might thus adjust the treatment time of luggage after bed bug encounters to a satisfactory level, and may consequently make a contribution to limit unwanted dispersal and the increasing bed bug problem.

## Supporting information


**Figure S1** The experimental design of cold treatment of Cimex lectularius 5th instar nymphs and adults. The upper part describes the complete experiment, and the lower parts are detailed descriptions of the actual temperature regimens during cold treatment (exemplified by the ‐5°C) with fluctuating (left) and constant (right) treatments.

**Figure S2.**. The temperature‐time combinations tested on Cimex lectularius 5th instar nymphs and adults.
