# Supplementary material for: Mortality, fecundity and development among bed bugs (Cimex lectularius) exposed to prolonged, intermediate cold stress
Source: Pest Manag Sci. 2017 Jan 31;73(5):838–43. doi: 10.1002/ps.4504 (PMC5396144; doi:10.1002/ps.4504)
Supplement: Supplementary file 2 — Figure S2.. The temperature‐time combinations tested on Cimex lectularius 5th instar nymphs and adults. [file PS-73-838-s002.docx]

| **1 week** | | **2 weeks** | | **3 weeks** | |
| --- | --- | --- | --- | --- | --- |
| *Constant* | *Fluctuating* | *Constant* | *Fluctuating* | *Constant* | *Fluctuating* |
| **0**°C | **0**°C (4 to -2)^a^ | **0**°C | **0**°C (4 to -2) | **0**°C | **0°C** (4 to -2) |
| **-5**°C | **-5°C** (-1 to -7) | **-5**°C | **-5°C** (-1 to -7) | **-5**°C | **-5°C** (-1 to -7) |
| **-7**°C | **-7**°C (-3 to -9) | **-7**°C | **-7**°C (-3 to -9) | **-7**°C | **-7°C** (-3 to -9) |
| **-10°C** | **-10°C** (-6 to-12) | **-10°C** | **-10°C** (-6 to-12) | **-10°C** | **-10°C** (-6 to-12) |
| **-15**°C | - | **-** | - | - | - |

^a^ The fluctuating treatments varied between the 2 extremes (in brackets) for 8 and 16 hours in a 24-hour cycle so as to provide the same average temperature as that of the constant treatment.
